# Systematic Identification of Rhythmic Genes Reveals *camk1gb* as a New Element in the Circadian Clockwork

**DOI:** 10.1371/journal.pgen.1003116

**Published:** 2012-12-20

**Authors:** Adi Tovin, Shahar Alon, Zohar Ben-Moshe, Philipp Mracek, Gad Vatine, Nicholas S. Foulkes, Jasmine Jacob-Hirsch, Gideon Rechavi, Reiko Toyama, Steven L. Coon, David C. Klein, Eli Eisenberg, Yoav Gothilf

**Affiliations:** 1 Department of Neurobiology, George S. Wise Faculty of Life Sciences, Tel Aviv University, Tel Aviv, Israel; 2 Sagol School of Neuroscience, Tel Aviv University, Tel Aviv, Israel; 3 Institute of Toxicology and Genetics, Karlsruhe Institute of Technology, Eggenstein, Germany; 4 Cancer Research Center, Sheba Medical Center, Tel Hashomer and Sackler School of Medicine, Tel Aviv University, Tel Aviv, Israel; 5 Laboratory of Molecular Genetics, Eunice Kennedy Shriver National Institute of Child Health and Human Development, National Institutes of Health, Bethesda, Maryland, United States of America; 6 Program in Developmental Endocrinology and Genetics, Eunice Kennedy Shriver National Institute of Child Health and Human Development, National Institutes of Health, Bethesda, Maryland, United States of America; 7 Raymond and Beverly Sackler School of Physics and Astronomy, Tel Aviv University, Tel Aviv, Israel; Stanford University School of Medicine, United States of America

## Abstract

A wide variety of biochemical, physiological, and molecular processes are known to have daily rhythms driven by an endogenous circadian clock. While extensive research has greatly improved our understanding of the molecular mechanisms that constitute the circadian clock, the links between this clock and dependent processes have remained elusive. To address this gap in our knowledge, we have used RNA sequencing (RNA–seq) and DNA microarrays to systematically identify clock-controlled genes in the zebrafish pineal gland. In addition to a comprehensive view of the expression pattern of known clock components within this master clock tissue, this approach has revealed novel potential elements of the circadian timing system. We have implicated one rhythmically expressed gene, *camk1gb*, in connecting the clock with downstream physiology of the pineal gland. Remarkably, knockdown of *camk1gb* disrupts locomotor activity in the whole larva, even though it is predominantly expressed within the pineal gland. Therefore, it appears that *camk1gb* plays a role in linking the pineal master clock with the periphery.

## Author Summary

The circadian clock is a molecular pacemaker that drives rhythmic expression of genes with a ∼24-hour period. As a result, many physiological processes have daily rhythms. Many of the conserved elements that constitute the circadian clock are known, but the links between the clock and dependent processes have remained elusive. With its amenability to genetic manipulations and a variety of genetic tools, the zebrafish has become an attractive vertebrate model for the quest to identify and characterize novel clock components. Here, we take advantage of another attraction of the zebrafish, the fact that its pineal gland is the site of a central clock which directly receives light input and autonomously generates circadian rhythms that affect the physiology of the whole organism. We show that the systematic design and analysis of genome-wide experiments based on the zebrafish pineal gland can lead to the discovery of new clock elements. We have characterized one novel element, *camk1gb*, and show that this gene, predominantly expressed within the pineal gland and driven by the circadian clock, links circadian clock timing with locomotor activity in zebrafish larvae.

## Introduction

All organisms demonstrate a wide variety of physiological, biochemical and behavioral daily rhythms that are driven by intrinsic oscillators, known as circadian clocks. These oscillators work in harmony with the 24 hours periodic changes in environmental conditions. The maintenance and synchronization of the circadian oscillator constitute an adaptive advantage that is evident from the high evolutionary conservation of the circadian system [1].

The current dogma regarding the mechanism of a circadian oscillator is based on positive and negative transcriptional-translational feedback loops with a time period of ∼24 hours. According to this model, in vertebrates, a positive transcription complex, the CLOCK:BMAL heterodimer, activates transcription of the negative clock components, *period* (*per*) and *cryptochrome* (*cry*) genes, by binding to E-box elements in their promoters. Negative feedback is achieved by PER:CRY heterodimers that enter the nucleus and suppress their own transcription by physically associating with the CLOCK:BMAL heterodimers, thus closing the feedback loop. Transduction of circadian information from this core oscillator is accomplished by the rhythmic activation of clock-controlled output genes, which in turn regulate downstream processes [2]. Several output genes contribute to the accuracy and stability of the oscillator. These encode transcriptional regulators, which constitute accessory loops that feedback to the core loops, or post-translational modifiers of core clock proteins. This system regulates diverse biochemical pathways which are thought to ultimately lead to the wide variety of physiological and behavioral daily rhythms [2]. In recent years, several factors which receive information from the core clock and schedule various output pathways have been revealed [3]–[5]. It is likely that these factors do not account for all core clock regulated processes. Accordingly, the quest for additional mediators is ongoing.

As is the case for many other non-mammalian vertebrates, the zebrafish pineal gland is considered to function as a master circadian clock organ; it is photoreceptive and houses a self-sustained autonomous clock that drives the daily rhythm in the synthesis of melatonin, an important endocrine element of the vertebrate circadian system [6], [7]. In addition to this hormonal output, neurons of the pineal gland project to brain targets [8]. Through these neuronal and hormonal signals, the pineal gland is thought to convey information regarding the circadian cycle to physiological and behavioral processes [9]. Therefore, this tissue has been extensively studied with the intention of elucidating the molecular components of the core clock [10]–[13]. However, the exact pathways which link the core molecular oscillator within the pineal gland to rhythmic physiological and behavioral processes of the entire organism remain largely unknown.

DNA microarray technology is a powerful tool, extensively used to identify circadian changes in the abundance of transcripts (i.e., circadian genes) throughout the animal kingdom. Using this approach in various tissues including the pineal gland, it has been demonstrated that the circadian clock controls groups of genes linked to a large number of molecular and cellular functions [14]–[17]. Surprisingly, different studies show only a moderate level of overlap among the genes identified as circadian in the same tissue from different species and sometimes even in the same species [18], [19]. These discrepancies could be explained by true biological differences or by the use of different experimental procedures and data analysis methods [18]. However, these discrepancies can also be partially attributed to the inherent limitations of DNA microarray technology, for example cross-hybridization of probe sets [20]. Improvement of circadian profiling is now feasible using next-generation sequencing technology to perform RNA-seq. This method is superior because it provides an unbiased measurement of the entire transcriptome without being restricted to only a subset of genes interrogated by the probe sets on a microarray chip [21]. However, methods to minimize errors and biases generated by RNA-seq are still being developed [22].

Here, we have systematically identified circadian genes in the zebrafish pineal gland, employing both DNA microarrays and RNA-seq; these findings were subsequently confirmed using independent quantitative assays. As described below, this strategy has resulted in the identification of a new element in the circadian timing system that possibly links the core clock with rhythmic locomotor activity in the zebrafish.

## Results

### Systematic identification of circadian genes in the pineal gland

**Figure 1:**
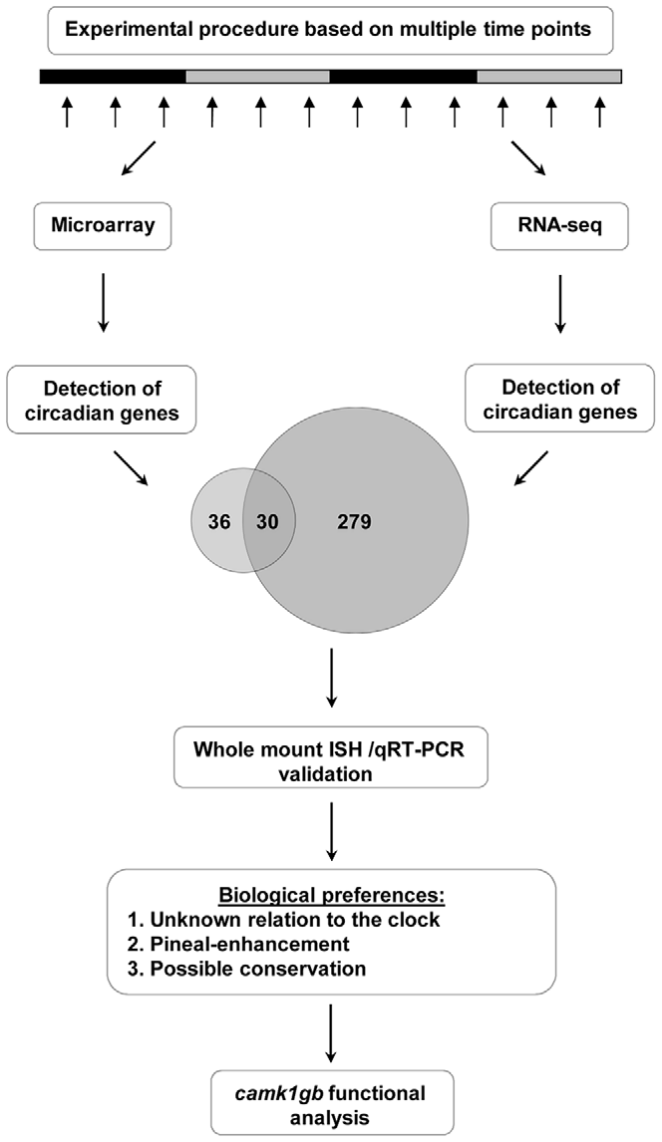
A step-by-step flow-chart of the systematic identification of pineal gland circadian genes. This procedure ultimately led to the detection of a new player, *camk1gb*, within the clock system.

Aiming at identifying circadian genes, we extracted RNA through two daily cycles from pineal glands of zebrafish previously adapted to 24 hours light dark cycles and then transferred to constant darkness during sampling (Figure 1 and Methods). This procedure was repeated twice with different sets of fish. The mRNA from the first experiment was quantified using Affymetrix DNA microarrays whereas the mRNA from the second experiment was quantified using RNA-seq (Methods). The data obtained from the DNA microarrays and RNA-seq analysis was subjected to Fourier analysis (Methods and Levy et al. [23]). Demanding 90% true-positives rate, the DNA microarrays and RNA-seq analysis resulted in 112 circadian probe-sets and 309 circadian genes, respectively (Tables S1 and S2). Altogether, 82 out of the 112 probe-sets identified by the DNA microarray method reliably represent zebrafish mRNAs from GenBank, 66 of which are well-annotated NCBI mRNA reference sequences collection (RefSeq) genes (Table S1). In the analysis of the RNA-seq, only sequencing reads that were aligned to genomic locations of RefSeq genes were used (Methods).

The larger number of circadian genes identified using RNA-seq is due in part to the greater number of genes measured; only about half of the RefSeq genes are represented on the DNA microarray (7634 out of 14263 RefSeq genes). In addition, RNA-seq has a higher detection power due to better accuracy in expression measurement [24]: out of the 309 RefSeq genes identified using RNA-seq, 180 were represented on the DNA microarray but only 30 of them (17%) were identified as circadian. In contrast, about half (30 out of 66) of the RefSeq genes detected as circadian using DNA microarrays were also identified as being circadian using RNA-seq, demonstrating better accuracy of the RNA-seq and overall reasonable agreement between the two methods. Notably, the 309 circadian genes are enriched with pineal-enhanced genes, i.e. genes with higher expression in the pineal gland compared to other tissues (3 out of the 29 pineal-enhanced genes identified in [25], P-value<0.05, binomial cumulative distribution) and with genes which were previously reported to have notable expression in the pineal gland (28 out of the 485 genes mentioned in the ZFIN database [26], P-value<10^−3^, binomial cumulative distribution).

### A comprehensive view of the expression pattern of known clock components

**Figure 2:**
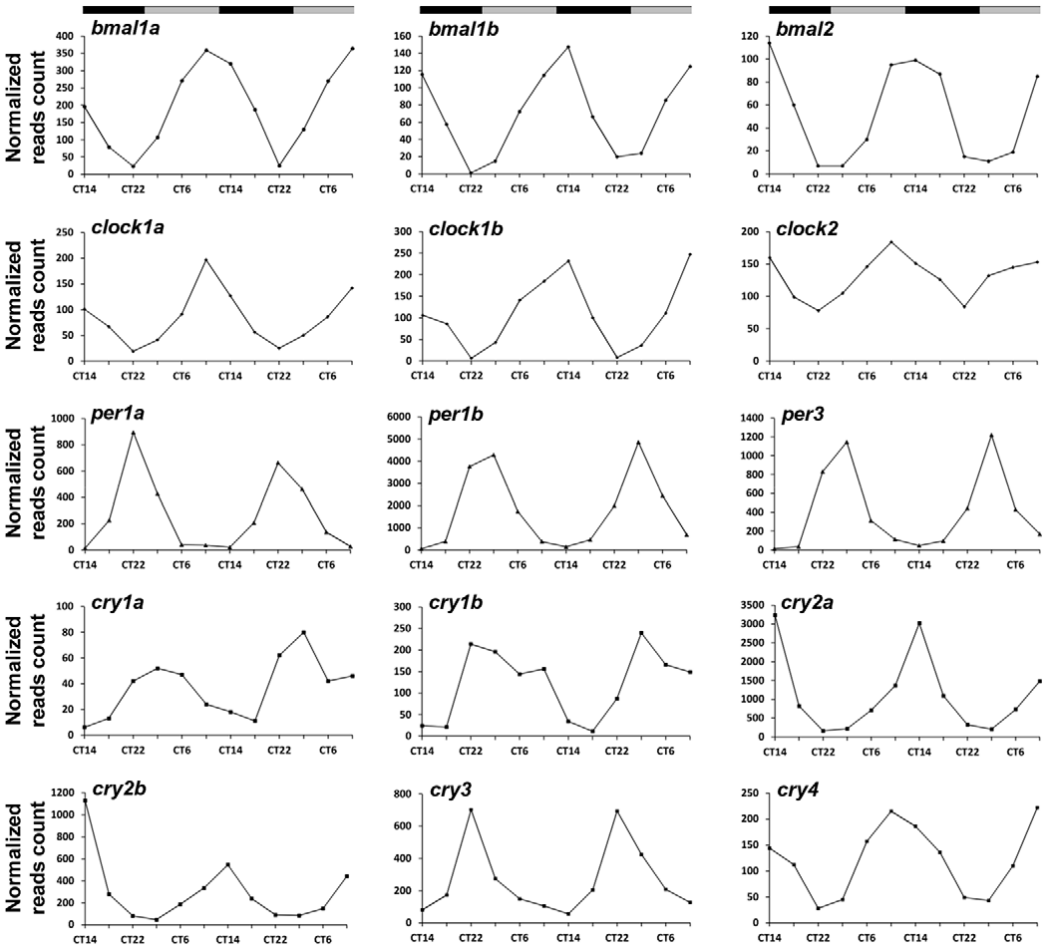
Circadian profiles of known core clock genes which were identified in the zebrafish pineal gland by the RNA–seq analysis. CT = circadian time. Gray and black bars represent subjective day and subjective night, respectively.

Nearly all (15 out of 16) of the known zebrafish core clock genes were identified as circadian in the RNA-seq analysis (Figure 2 and Table S3). The only exception was *per2* which is known to be light-induced in the pineal gland and not circadian under constant darkness [27]. Notably, the RNA-seq analysis is in agreement with the reported phases of 14 core clock genes (Table S3). Similarly, most of the genes (12 out of 14) that are considered to form accessory loops of the molecular circadian oscillator were identified as circadian and their phases are in agreement with previous experimental data (Table S4). In accordance, functional annotation analysis using DAVID [28] reveals the pathway ‘Circadian rhythms’ as significantly enriched (Benjamini-Hochberg adjusted P-value<1e-17) within the identified circadian genes (Table S5 and Methods). As only a portion of the zebrafish genes are represented on the Affymetrix DNA microarray it is reasonable that the list of circadian genes revealed by RNA-seq is larger. Nevertheless, 8 known clock genes (3 core clock and 5 accessory loops-related) are included within the 82 circadian genes identified in the DNA microarray experiment (Tables S3 and S4), thereby providing evidence that other results generated by this analysis are reliable. Importantly, the extensively studied pineal gland clock-controlled gene, *aanat2* (*arylalkylamine N-acetyltransferase*) [29], was identified using both the DNA microarray and RNA-seq analyses. Accordingly, it is clear that these methods provide an informative view of circadian changes in the abundance of transcripts in the zebrafish pineal gland.

### Selecting potential candidates that relay circadian timing information

We aimed to characterize new regulators of the pineal master clock or new mediators relaying circadian information to downstream processes. The genes detected using RNA-seq and DNA microarray analysis can serve as a basis for this quest. Thirty genes that were identified using both these two independent methods were considered for further functional analysis (Table 1). Notably, about one third of these genes (9 out of 30) were previously reported as core clock or clock-controlled genes (Table 1). In addition, qRT-PCR and quantitative whole mount *in situ* hybridization (ISH) were performed on selected genes as a validation procedure (Methods); as expected, nearly all (8 out of 9) of the tested genes were indeed validated as circadian, showing similar phases to those identified by the DNA microarray and RNA-seq data (Figure S1, Table 1 and Methods). The use of the two independent genome-wide methods, the re-discovery of previously reported core clock genes and the validation procedure, confirmed that the concise list (Table 1) represent *bona fide* circadian genes.

**Table 1:** Genes detected as circadian using both DNA microarray and RNA–seq.

Genes name	RefSeq number	Known zebrafish clock-controlled genes	Pineal-enhanced expression	Experimental validation
*aanat2*	NM_131411	[29]	Yes	[29]
*ankhb*	NM_194370			
*arg2*	NM_199611		Yes	data from ZFIN
*bhlhe40*	NM_212679	[66]	Yes	Whole mount ISH
*bhlhe41*	NM_001039107	[66]	Yes	Whole mount ISH
*bmal1a*	NM_131577	[67]		
*bmper*	NM_001020487		Yes	[68],[69]
*camk1gb*	NM_200829		Yes	Whole mount ISH
*cdh2*	NM_131081			qRT-PCR
*cry2a*	NM_131791	[70]		
*cry3*	NM_131786	[70]		
*dbpa*	NM_001197060	[71]	Yes	[71]
*dhrs9*	NM_199609			qRT-PCR
*fam73a*	NM_001006090			
*fbxo25*	NM_205724			qRT-PCR
*guk1b*	NM_200724		Yes	data from ZFIN
*ldha*	NM_131246			
*mid1ip1l*	NM_213439			
*ndrg1b*	NM_200692		Yes	Whole mount ISH
*nfil3-5*	NM_001197058	[71]	Yes	[71]
*nr1d2b*	NM_131065	[72]	Yes	Whole mount ISH
*rhoub*	NM_001017784			
*rimkla*	NM_001004554			
*sh3gl2*	NM_201116			Whole mount ISH
*slc38a4*	NM_001005944			
*tcp11l2*	NM_213020			
*tob1b*	NM_212974			
*zgc:152863*	NM_001080000			
*zgc:153018*	NM_001076639			
*zgc:193593*	NM_001128717			

For further functional analysis we focused on genes that were not previously connected to the core clock or the core clock accessory loops (Tables S3 and S4). Studying pineal-enhanced genes can aid in elucidating the role of the master clock in coordinating downstream circadian rhythms. We thus selected genes from the concise list based on their expression pattern, focusing only on those showing enhanced expression in the pineal gland. This was determined using whole mount ISH in larvae (Table 1 and Figure S1). Of the previously unreported genes from the concise list, 5 fulfilled the above requirements: *camk1gb*, *guk1b*, *arg2*, *bmper* and *ndrg1b* (Table 1). Of these, we chose to focus on *camk1gb* (*calcium/calmodulin-dependent protein kinase IGb*). The mammalian *Camk1g* is a member of a larger family of calcium/calmodulin-dependent protein kinases, the CaMKI family. Like other members of this family, *Camk1g* requires both calcium/calmodulin and phosphorylation by CaMKK for its full activation [30]. *camk1gb* is one of two zebrafish homologs of the mammalian *Camk1g*. Our pineal gland RNA-seq data shows that the expression levels of the other paralog, *camk1ga*, are similar to those of *camk1gb*. Yet, it was not found to be expressed in a circadian manner (Table S2). Interestingly, like *camk1gb*, the mammalian *Camk1g* was reported to exhibit enhanced and circadian expression in the rat pineal gland [16], [31], suggesting a conserved role in the pineal clock or in other aspects of pineal function.

### *camk1gb* spatiotemporal expression

**Figure 3:**
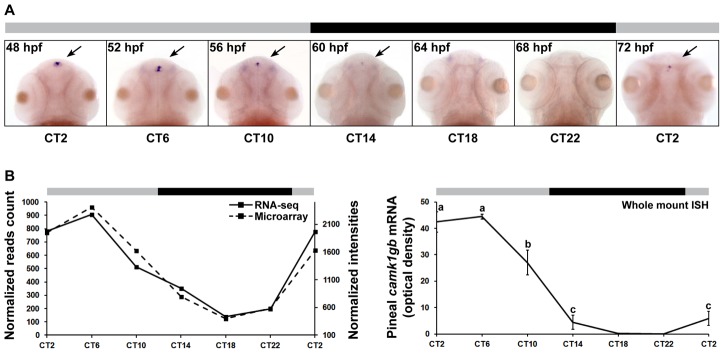
*camk1gb* spatio-temporal expression. A) Rhythmic expression of *camk1gb* exclusively in the pineal glands (indicated by black arrows) of 48–72 hpf embryos as detected by whole mount ISH under constant darkness. B) Rhythmic expression of *camk1gb* in the zebrafish embryo (right) is correlated with the RNA-seq (solid line, left vertical axis) and the microarray data (dashed line, right vertical axis) from the adult (left). Correlation coefficients between the whole mount ISH results and the data obtained by microarrays and RNA-seq were determined by Pearson correlation (r = 0.89 and 0.77, respectively). For whole mount ISH, statistical differences in mRNA levels were determined by one-way ANOVA followed by a Tukey test (P-value<0.05). Error bars represent SE (n = 10–15). CT = circadian time. Gray and black bars represent subjective day and subjective night, respectively.

Whole mount ISH of larvae clearly reveals enhanced pineal expression of *camk1gb* (Figure 3a and Figure S2). Importantly, the circadian expression pattern in the pineal gland of larvae, characterized by an expression peak at CT6, is similar to the profile in the pineal gland of adults: Pearson's correlations of 0.89 and 0.77 to the DNA microarrays and RNA-seq profiles, respectively (Figure 3b). This similarity may suggest that the temporal profile of *camk1gb* has a functional significance starting from early life stages until adulthood.

### *camk1gb* knockdown disrupts circadian larval locomotor activity

**Figure 4:**
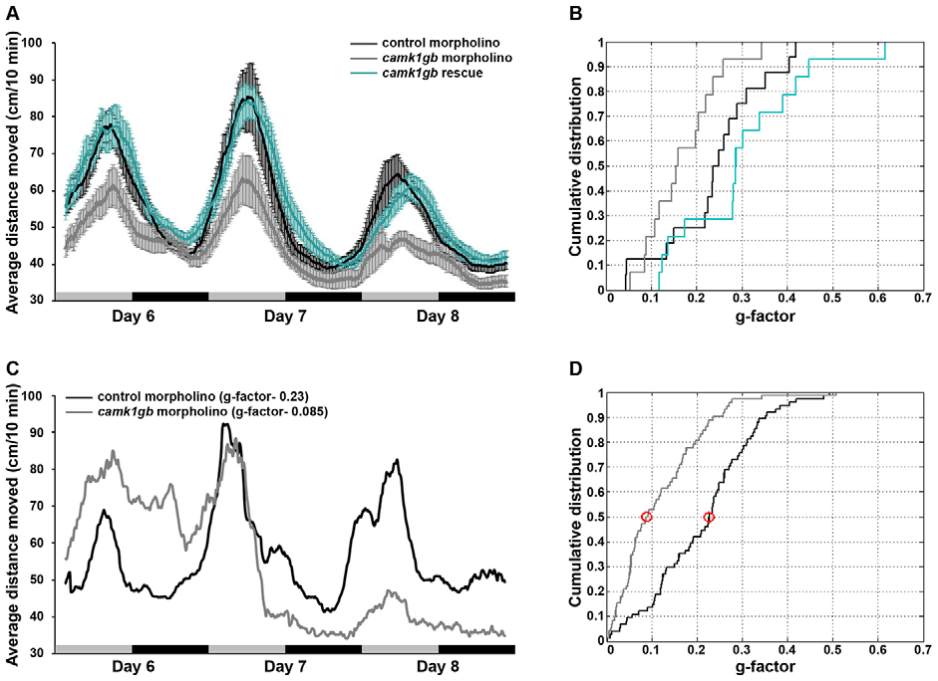
The effect of *camk1gb* knockdown on larval locomotor activity rhythms. A) Average locomotor activity of 6–8 dpf larvae injected with either control morpholino (black trendline), *camk1gb* morpholino (gray trendline) or co-injected *camk1gb* morpholino together with *camk1gb* mRNA (green trendline), under constant dim-light. Activity was measured as the average distance moved for time units of 10 min and smoothed using moving average. Error bars represent SE (n = 16). Black and gray horizontal boxes represent subjective night and day, respectively. B) Cumulative distribution of g-factor values for each group. Significant differences in the g-factor distribution were revealed between the control morpholino and *camk1gb* morpholino treated groups as well as between the rescue and *camk1gb* morpholino injected groups (Kolmogorov-Smirnov test, P-value<0.05 and <0.01, respectively). C) Representative activity profiles of control morpholino (black curve) and *camk1gb* morpholino (gray curve) injected larvae which correspond to the median g-factor of each group. The median g-factor values are marked by red circles in (D). D) Significant differences in the cumulative distribution of g-factor values between control morpholino (black curve) and *camk1gb* morpholino (gray curve) injected groups (n = 75), generated from the analysis of four similar experiments (Kolmogorov-Smirnov test, P-value<10^−5^).

We examined the effect of *camk1gb* on clock-regulated zebrafish behavior. Zebrafish larvae exhibit robust circadian locomotor activity with highest activity during the subjective day [32], [33]. To determine whether *camk1gb* is required for normal circadian locomotor activity, embryos were injected with either control morpholino or *camk1gb* morpholino (Methods). *camk1gb* morpholino treatment results in the inclusion of an intron within the mRNA coding sequence and the consequent introduction of a premature stop codon (Figure S3 and Text S1). Strikingly, *camk1gb* knockdown significantly disrupted the circadian activity pattern (Figure 4a and Methods). This experiment was repeated 4 times using a total of 75 larvae injected with *camk1gb* morpholino and 75 larvae injected with control morpholino (Methods). The disrupted circadian activity pattern is also evident when analyzing individual larvae using Fourier analysis (Figure 4b, Figure 4c, Figure 4d and Methods). Only 8/75 of the *camk1gb* knockdown larvae have shown a 24 h-period signal that surpasses the median signal for the 75 control larvae (Figure 4d and Methods). Furthermore, we tracked locomotor activity levels at abrupt light to dark transitions [34]; both control and *camk1gb* knockdown groups showed similar levels of locomotor activity, indicating that *camk1gb* knockdown does not impair larval movement abilities (Figure S4 and Methods). Lastly, a rescue experiment, in which *camk1gb* mRNA was co-injected along with the *camk1gb* morpholino, restored normal circadian activity thereby demonstrating the specificity of the injected morpholino (Figure 4a and Methods). The success of the rescue experiment is remarkable given that the injected mRNA is likely to restore the levels of *camk1gb* but less likely to restore its rhythmic expression. Therefore, it seems that sufficient expression levels of *camk1gb* are necessary for proper circadian rhythms of locomotor activity. Alternatively, it is possible that posttranscriptional regulation may restore the rhythmic expression pattern of the protein, thereby contributing to the success of the rescue experiment.

### *camk1gb* is required for the proper rhythmic transcription of *aanat2*

**Figure 5:**
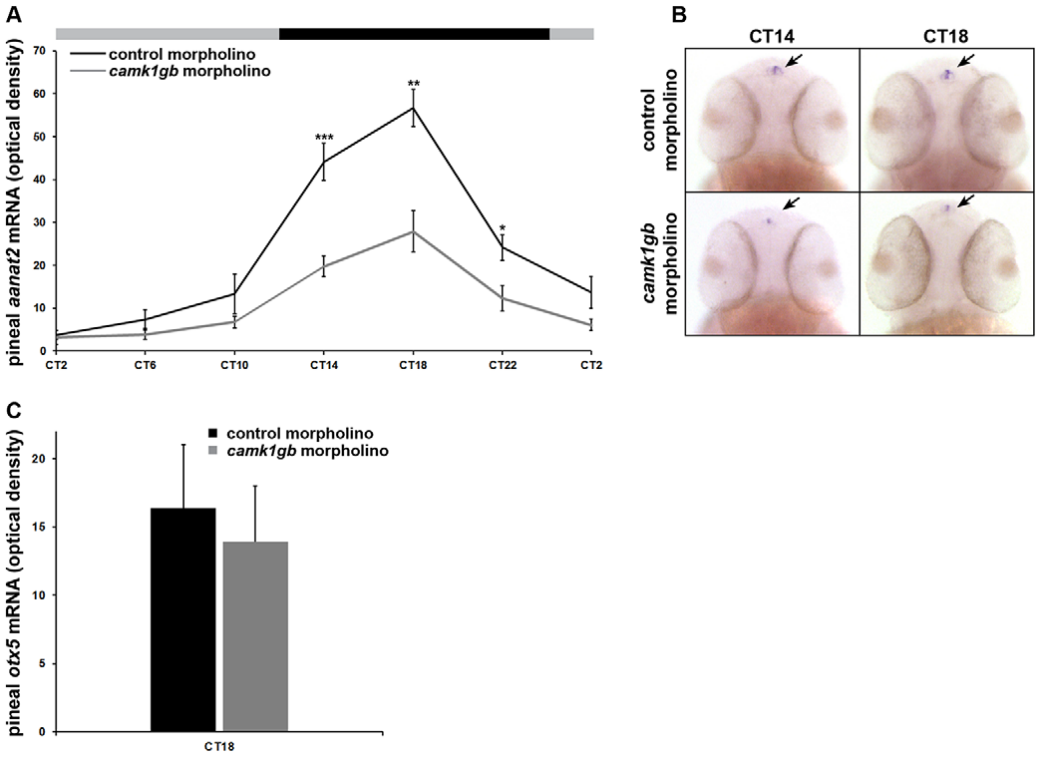
Effect of *camk1gb* knockdown on pineal *aanat2* and *otx5* mRNA rhythms. Zebrafish embryos injected with either control morpholino (black line) or *camk1gb* morpholino (gray line) were subjected to DD during their third day of development and pineal *aanat2* and *otx5* mRNA levels were determined by whole mount ISH. A) Embryos were sampled at 4-hr intervals for *aanat2*. Statistical differences in *aanat2* mRNA levels between the control morpholino and *camk1gb* morpholino injected embryos were determined by two-tailed *t*-test with Bonferroni correction (* P-value<0.05, ** P-value<0.01, *** P-value<0.001). B) Whole-mount ISH for *aanat2* in the heads (dorsal views) of representative larvae from each group, at CT14 and CT18. Arrows indicate pineal *aanat2* mRNA expression. C) *otx5* expression at CT18. Error bars represent SE (n = 10–15). CT = circadian time.

The AANAT enzyme drives the rhythmic production of melatonin [35]. Zebrafish pineal *aanat2* transcription exhibits a robust circadian rhythm that begins at 2 days post-fertilization [29], [36]. The transcription of *aanat2* is tightly regulated by the core molecular oscillator [29] as well as other transcription factors [37]. Importantly, *camk1gb* knockdown significantly reduced (Student's *t*-test, Bonferroni corrected P-value<0.05) the amplitude of the *aanat2* expression rhythm by half as detected by whole mount ISH (Figure 5a, Figure 5b and Methods). *camk1gb* knockdown did not affect normal pineal gland development as indicated by whole mount ISH for *otx5* [13] (Figure 5c). These results demonstrate that *camk1gb* is not necessary for *aanat2* to be transcribed but is involved in the physiological regulation of the rhythmic transcription of pineal *aanat2*.

### *camk1gb* knockdown effects are not due to core clock disruption

The disruption of the circadian locomotor activity and the reduction in *aanat2* rhythmic expression may suggest that *camk1gb* is a previously unrecognized regulator of the core clock. This notion is in line with findings showing that distant CaMK family members can modulate core clock genes [38]–[41]. We reasoned that if indeed *camk1gb* is important for normal core clock function, the effect of its knockdown will be manifested in the expression patterns of additional circadian genes. Hence, we tested the effect of *camk1gb* knockdown on the expression levels of 3 additional pineal-enhanced clock-controlled genes (*sh3gl2, opn1lw1* and *ndrg1b*) and 2 pineal-enhanced core clock accessory loops genes (*dec1* and *dec2*) using whole mount ISH at the peak and the nadir of their rhythm (Methods). However, the expression pattern of the tested genes was not significantly affected by *camk1gb* knockdown (Figure S5). Accordingly, over-expression of *camk1gb* in the zebrafish cell line, Pac-2, did not disrupt the core clock as indicated by examining the promoter activity of the core clock marker gene, *per1b* (Figure S6 and Methods). Thus, *camk1gb* knockdown affects circadian locomotor activity and *aanat2* expression levels without affecting the core clock.

## Discussion

In this study we set out to identify new molecular elements that affect the circadian timing system, either directly through the core clock or indirectly by relaying timing information from the core clock to downstream processes. It was previously demonstrated that identification of circadian genes using DNA microarrays can lead to the discovery of previously unrecognized clock components [42]. We reasoned that by using RNA-seq a significant improvement in the number of the detected circadian genes can be achieved, with better detection reliability. However, this new technique introduces non-trivial problems into data analysis and can also bring about biases in genes' quantification [43]–[46]. These hurdles can be overcome by integrating several experimental procedures and employing rigorous and stringent data analysis. Therefore, circadian genes were systematically identified using two independent high-throughput methods, RNA-seq and DNA microarray analyses, followed by computational analysis and extensive *in vivo* validations.

In this study we focused on the zebrafish pineal gland for two main reasons: 1) most of the molecular clock components are likely to be functional in this autonomous clock tissue. Indeed, the expression of nearly all the core clock genes was found to be circadian in this tissue (Figure 2). 2) By focusing on pineal genes the link between a master clock and peripheral tissues may be elucidated. Indeed, we demonstrated that *camk1gb*, which is pineal-enhanced, controls circadian downstream processes within the pineal gland and in the entire animal (i.e. locomotor activity).

The link between *camk1gb* and circadian locomotor activity is intriguing, especially since *camk1gb* is a pineal-enhanced gene. Although the possibility that *camk1gb* affects circadian locomotor activity through its low expression in structures outside the pineal gland cannot be ruled out, the enhanced and circadian expression of this gene in the pineal gland (Figure 3) suggests otherwise. At least two possible mechanisms might explain how *camk1gb* relays circadian timing information from the pineal gland. One is by regulating melatonin secretion: *camk1gb* knockdown caused a 50% reduction in the night-time expression of *aanat2*, the key enzyme in melatonin production [35]. Melatonin administration has a profound effect on locomotor activity rhythms in many organisms including zebrafish [47], [48]. Therefore, it is tempting to speculate that disruption of melatonin levels leads to the observed alteration in circadian locomotor activity. However, the role of endogenous melatonin rhythms in the maintenance of normal locomotor activity rhythms in zebrafish is still not fully understood and therefore warrants further investigation. A second possible mechanism is by modulating neuronal innervations between the pineal gland and deeper brain regions [8]. Mammalian *Camk1g* is known to coordinate neuronal morphogenesis. This CaMKI isoform is a membrane-anchored protein, abundant in neurons, which mediates dendritic and axonal outgrowth of neurons in culture [49], [50]. In teleost fish, the majority of pineal photoreceptor cells form contacts with postsynaptic neurons which send processes to the brain. A fraction of the pineal photoreceptor cells possess long axons that project directly to the brain [9]. Interestingly, we find that *camk1gb* is indeed expressed within photoreceptors (Figure S7 and Methods). Taken together, these observations point to the possibility that *camk1gb* is required for the transmission of circadian timing information from the central clock in the pineal gland to the brain.

We provide evidence that *camk1gb* regulates the transcription of *aanat2*. Naturally, understanding this regulatory mechanism is of interest. As is the case for most members of the CaMKI family, it was previously demonstrated *in vitro* that the transcription factor cAMP-response element-binding protein (CREB) is one of the phosphorylation targets of the mammalian homolog, *Camk1g* [30]. It was also reported that *Camk1g* regulates CREB-mediated transcription [51]. Interestingly, phosphorylated CREB regulates the transcription of *Aanat* in mammals [52], [53], and possibly in other vertebrates [37]. Therefore, it is possible that *camk1gb* modulates the levels of phosphorylated CREB which in turn affects the transcription levels of *aanat2*. We note however that the *camk1gb* expression peak is in mid-day whereas *aanat2* peaks in mid-night (Figure 3 and Figure 5). A reasonable explanation may be an expected time lag between *camk1gb* transcription and the appearance of its translated product. An alternative mechanism involves an indirect effect in which *camk1gb* regulates the pineal core clock and thereby controls *aanat2* transcription. However, we found no evidence that *camk1gb* knockdown affects the core clock mechanism (Figures S5 and S6). Nevertheless, based on findings showing that distant members of the CaMK family can modulate core clock genes [38]–[41], further examination of this possibility in tissues other than the pineal gland is justified.

A comprehensive view of the pineal gland circadian transcriptome allows a dissection of functions that are clock-related inside the pineal gland. As expected, genes that belong to the ‘Circadian rhythms’ pathway are significantly enriched within the pineal gland circadian transcriptome (Table S5 and Methods). The ‘Glycolysis’ and ‘Pyruvate metabolism’ processes are also significantly enriched (Table S5), including 4 circadian enzymes out of the 10 required for glycolysis (Table S6a). Recent studies have revealed a close link between the core clock and metabolism that is mediated by REV-ERB transcription factors [3], [4]. In particular, *rev-erb* couples glycolysis/gluconeogenesis with the core clock in the mouse liver [54]. Our findings suggest that links between glycolysis and the core clock are not restricted to the liver but may be present in other tissues. Another interesting function which was found to be enriched is ‘Oxidation reduction’ (Table S5). Twenty-five circadian genes belong to this pathway including *catalase* and 7 different cytochromes P-450 (Table S6b). Several genes in the ‘Oxidation reduction’ pathway were shown to be clock-regulated [55]. In zebrafish cells, *catalase* has been implicated in the light-dependent transcription of clock genes [56]. Our data suggest that the link between this pathway and the clock system may be more general and includes both central and peripheral clock tissues.

We have constructed a database that contains many interesting candidates for future investigation in the context of either regulating the core clock or in linking of the core clock to downstream pathways. We have focused on *camk1gb* and showed that this gene is rhythmically expressed in the pineal gland and affects daily rhythms of behavior. In mammals, several genes which connect the master clock to downstream circadian locomotor activity have been discovered [57]. They all share in common the following characteristics: 1. Rhythmic expression in the master clock (which is the suprachiasmatic nucleus in mammals). 2. Alterations in their levels disrupt circadian locomotor activity. Since *camk1gb* shares these properties and regulates *aanat2*, and therefore possibly melatonin, we suggest that this gene serves to connect the master clock with circadian locomotor activity in zebrafish.

For over a decade, zebrafish seemed to represent an ideal vertebrate model for the quest to identify and characterize novel clock components [10]. However, with the exception of one study [58], no novel clock components have been identified to date using this model. Instead, the zebrafish has been used to further characterize clock components that were previously identified in mammals. Here, we have demonstrated that the design and analysis of systematic high-throughput experiments based on zebrafish can lead to the discovery of new clock elements.

## Methods

### Ethics statement

All procedures were approved by the Tel Aviv University Animal Care Committee and conducted in accordance with the council for experiments on animal subjects, Ministry of Health, Israel.

### DNA microarrays and RNA–seq experimental design

The experimental procedure for the DNA microarrays experiment was performed as follows. Adult (0.5–1.5 years old) transgenic zebrafish, *Tg(aanat2:EGFP)^Y8^*, which express enhanced green fluorescent protein (EGFP) in the pineal gland under the control of the *aanat2* regulatory regions, were used [29]. Fish were raised under 12-hr light∶12-hr dark (LD) cycles, in a temperature controlled room, and transferred to constant darkness (DD) for tissue collection. Fish were anesthetized in 1.5 mM Tricane (Sigma), sacrificed by decapitation, and pineal glands were removed under a fluorescent dissecting microscope. Starting from circadian time (CT) 14, pineal glands were collected at 4-hr intervals for 48 hours (12 time points identified as CT 14, 18, 22, 2, 6, 10, 14b, 18b, 22b, 2b, 6b and 10b). Pools of 12 (DNA microarray) or 20 (RNA-Seq) pineal glands were prepared at each time-point and total RNA was extracted using the RNeasy Lipid Tissue Mini Kit (QIAGEN), according to the manufacturer's instructions.

### DNA microarrays

Labeled RNA preparation and hybridization to DNA microarrays were performed according to the Affymetrix manual with the two-cycle target labeling protocol (http://www.affymetrix.com/support/downloads/manuals/expression_analysis_technical_manual_.pdf). A total of 12 Affymetrix DNA microarrays were hybridized with RNA-pools of pineal glands from 12 time points throughout two daily cycles. Each DNA microarray was normalized using Affymetrix GeneChip Operating Software (GCOS). The entire DNA microarray dataset, logarithmically transformed, was normalized using quantile normalization to guarantee that the distribution of probe intensities was the same in all the chips [59]. The microarray data was deposited to the Gene Expression Omnibus (GEO), under accession GSE41696.

### RNA–seq

Illumina TruSeq protocol was used to prepare libraries from RNA samples. Overall, 12 libraries (12 time points) were run on 2 lanes of Illumina HiSeq2000 machine using the multiplexing strategy of the TruSeq protocol (*Institute of Applied Genomics*). On average, ∼30 million paired-end reads were obtained for each library. The reads were 2×100 base pairs for 8 time points (CTs 22, 2, 6, 10, 18b, 2b, 6b, 10b) and 2×50 base pairs for the remaining time points (CTs 14, 18, 14b and 22b). TopHat [60] was used for aligning the reads against the zebrafish genome allowing only uniquely aligned reads and up to two mismatches per read. On average, 56% of the reads had unique alignment to the zebrafish genome. Reads aligned to the protein coding regions of known RefSeq genes were used. A custom script written in Perl was used to parse the output of TopHat, which is given in Sequence Alignment/Map (SAM) format (http://samtools.sourceforge.net/), and to convert it into raw number of reads aligned to each position in each RefSeq gene. The RefSeq genes information was obtained from the Table Browser of the UCSC genome browser (genome.ucsc.edu/) using the zebrafish Jul. 2010 (Zv9/danRer7) assembly. To avoid PCR duplicates, only paired-end reads that have unique start position in the genome in both pairs were used [61].

The quality of the sequencing libraries was assessed as described in Levin et al. [61] and the data was normalized using Quantile normalization (Text S1). We made sure that the normalization scheme properly corrects for different RNA levels and other technical differences between samples (Text S1). The sequencing data was deposited to the Sequence Read Archive (SRA), under accession SRA054264.

### Fourier analysis

The time-dependent signal was converted into a frequency-dependent signal using the Fast Fourier Transform (FFT). The extent to which the original signal is circadian was quantified by the ratio (‘g-factor’) of the power (squared amplitude) of the frequency which corresponds to 24 hr period to the sum of powers of all frequencies [23]. The higher the g-factor, the higher is the confidence that the transcript is circadian. We note that changing the definition of the g-factor by adding the powers of higher harmonics of the 24 hr period to the numerator, gave similar results compared to the use of the definition above. To determine the true-positive rate for a list of transcripts constructed using a given g-factor cut-off, permutation analysis was conducted as follows:

1. The time-dependent signals of each transcript were randomly shuffled.
2. FFT was performed and g-factor was calculated for each transcript.
3. The cumulative histogram of the g-factor values (ranging from 0 to 1) was calculated, resulting in the number of transcripts whose calculated g-factor is larger than a given value (‘random detection function’).
4. Steps (1)–(3) were repeated a thousand times and the averaged random detection function was calculated. This function estimates the number of false-positive detections of circadian genes for any given cutoff value of the g-factor.
5. Steps (2)–(3) were performed on the original data set. The resulting function, providing the number of transcripts exhibiting a g-factor larger than a given value in the real data, was termed the ‘detection function’.
6. For any given choice of cutoff for the g-factor, the difference between the detection function and the average random detection function estimates the number of true-positives in the list of transcripts constructed with this cutoff.

Finally, using the number of transcripts detected for a given g-factor (step 5) and the number of true-positives for a given g-factor (step 6), the true-positive rate as a function of the number of transcripts detected was calculated and further used to identify circadian genes with high accuracy (Figure S8). The procedure described here was implemented using in-house MATLAB (The Mathworks, Inc.) script.

### Gene ontology analysis

The 308 circadian RefSeq genes identified using the RNA-seq were analyzed to find over-represented molecular functions (Table S2), using the DAVID bioinformatics tools [28] and focusing on over-represented gene ontology (GO) categories and KEGG pathways [62], [63]. The DAVID's default zebrafish genes background was used. All the significantly enriched (Benjamini-Hochberg adjusted P-value<0.05) GO categories and KEGG pathways are presented in Table S5.

### Validation of the DNA microarrays and RNA–seq data

The temporal expression pattern of candidate genes was determined by whole mount ISH in zebrafish larvae or by quantitative RT-PCR in the adult pineal gland as previously described [12], [27] (Figure S1 and Text S1).

### Morpholino design and knockdown experiments

Morpholino experiments were conducted as previously described [11] (Text S1).

### Rescue experiments

Rescue experiments were conducted by co-injection of approximately 2 nl volume *of camk1gb* morpholino (1 mM) and *in vitro* transcribed *camk1gb* mRNA (100 ng/µl). The *camk1gb* protein-coding sequence was PCR-amplified with a KAPA HIFI PCR kit (KAPA Biosystems) using the same set of primers that was used for ISH experiments. The PCR products were cloned into a pCS2^+^ vector, linearized with *Not*I restriction enzyme and transcribed using the SP6 mMessage mMachine kit (Ambion), according to the manufacturer's instructions, to generate capped *camk1gb* mRNA.

### Locomotor activity experiments

Embryos were microinjected with either control morpholino, *camk1gb* morpholino or co-injected with *camk1gb* morpholino and *in vitro* transcribed *camk1gb* mRNA and kept under LD conditions for 3 days. On the fourth day post-fertilization, embryos were placed in 48-well plates in the observation chamber of the DanioVision Tracking System (Noldus Information Technology) and exposed, for acclimation, to two days under 12-hr light (3400 lux)∶12-hr dim light (40 lux) regime followed by 3 days of constant dim light. Live video tracking and analysis was conducted using the Ethovision 8.0 software (Noldus Information Technology). Activity was measured at days 6–8 post fertilization, as the distance moved by a larva in 10 min time bins (Figure 4). The activity record of each individual was subjected to Fourier analysis, and scored with a g-factor (see Methods section ‘Fourier analysis’). Significant differences in the g-factor distributions between the control and *camk1gb* morpholino treated groups (n = 75; combining 4 different experiments) as well as between the rescue and *camk1gb* morpholino treated groups (n = 16) were determined by the Kolmogorov-Smirnov test (Figure 4b and Figure 4d). The percent of larvae which are considered circadian depends on the g-factor used as the detection criteria, but for all values of g-factor cutoff tested (ranging between 0.05 and 0.3, Figure 4d), significantly more larvae are considered circadian in the control group.

To determine whether *camk1gb* knockdown impairs larval movement abilities, locomotor activity levels were tracked under abrupt light to dark transitions [34]. On day 6 post fertilization, control morpholino and *camk1gb* morpholino injected larvae (n = 24) were subjected to 3 dark flashes of 5 sec each during the light phase [34]. Activity was measured as the distance moved by each larva during the dark flash. No statistical difference was observed between the activity of the control morpholino and *camk1gb* morpholino injected groups (Student's *t*-test, P-value>0.2; Figure S4), indicating that the *camk1gb* morpholino does not impair larval movement abilities.

### Transfection of *camk1gb* into the Pac-2 cell line

Transient co-transfection of the Pac-2 cell line with *camk1gb* and *per1b*:luciferase constructs was performed as previously described [64] (Figure S6 and Text S1).

### Double ISH of *camk1gb* and *aanat2*:EGFP

For double fluorescence ISH we followed the protocol of Machluf and Levkowitz [65] (Text S1).

## Supporting Information

Figure S1 Whole-mount ISH and qRT-PCR validations. The circadian expression of several genes was validated using whole mount ISH on embryos at the age of 48–72 hours (A–E, right curves) and qRT-PCR on adult pineal glands (F–H, right curves). Left curves represent the circadian profile of each gene as obtained by RNA-seq (solid line, left vertical bar) and DNA-microarray (dashed line, right vertical bar). Representative pictures of embryos heads (dorsal view), subjected to whole mount ISH for *ndrg1b*, *dec1*, *dec2*, *reverbb2* and *sh3gl2* at CT2 and CT14, are presented at the rightmost side of A–E. The pineal gland is indicated by a red arrow. Different letters represent statistical differences in mRNA levels as determined by one-way ANOVA followed by a Tukey test (P-value<0.05). Note that *cdh2* was not validated as circadian (H). Error bars represent SE (n = 10–15). CT = circadian time. Gray and black bars represent subjective day and subjective night, respectively. Whole mount ISH validation of *camk1gb* is given in Figure 3.
(TIF)

Figure S2 *camk1gb* expression at later larval stages. *camk1gb* expression on days 4–6 post fertilization is enhanced in the pineal gland (pg) and expends to the retina (re), habenula (ha) and olfactory bulbs (olf) as detected by whole mount ISH.
(TIF)

Figure S3 PCR analysis of *camk1gb* following *camk1gb* morpholino injection. *camk1gb* morpholino injection changed the normal splicing of *camk1gb* mRNA, leading to an insertion of intron5 (right lane) which adds a premature stop codon. Injection of control morpholino had no effect on *camk1gb* splicing (left lane).
(TIF)

Figure S4 Locomotor activity levels under 3 dark flash stimuli. On day 6 post fertilization, control morpholino (solid line) and *camk1gb* morpholino (dashed line) injected larvae (n = 24) were subjected to 3 dark flashes (black, gray and blue lines) of 5 sec each during the light phase. Activity was measured as the average distance moved in 1 sec time bins. Error bars represent SE (n = 24). White and black horizontal boxes represent light phase and dark flash, respectively.
(TIF)

Figure S5 Effect of *camk1gb* knockdown on the expression of known clock-controlled genes. Zebrafish embryos injected with either control morpholino (black bar) or *camk1gb* morpholino (gray bar) were subjected to DD during their third day of development and sampled at the peak and the nadir of their rhythm. Pineal mRNA levels of *dec1, dec2, ndrg1l, sh3gl2 and opn1lw1* were determined by whole mount ISH. No statistically significant differences were observed (two-tailed *t*-test). Error bars represent SE (n = 10–15); CT = circadian time.
(TIF)

Figure S6 The effect of *camk1gb* over-expression on the core clock marker *per1b*. The zebrafish photosensitive Pac-2 cell line were transiently co-transfected with *camk1gb* and *per1b*:luciferase constructs. Bioluminescence was monitored under LD and DD conditions. No significant differences were found in the reporter construct expression as a result of *camk1gb* over-expression. White and black bars show the light and dark periods, respectively. Grey bars represent subjective day.
(TIF)

Figure S7 *camk1gb* expression in the adult pineal gland of transgenic zebrafish, *Tg(aanat2:EGFP)^Y8^*. Double fluorescent *in situ* hybridization for *egfp* mRNA (A, green) and *camk1gb* mRNA (B, red) in adult pineal glands, reveals co-expression of *camk1gb* and the *aanat2:EGFP* transgene (C, merged image). Scale bar  = 10 µm.
(TIF)

Figure S8 True-positive rate as a function of the number of circadian transcripts detected. A) DNA microarray experiment and B) RNA-Seq experiment. Red circles mark the size of the chosen list of transcripts.
(TIF)

Table S1 List of circadian genes using DNA Microarray.
(XLS)

Table S2 List of circadian RefSeq genes using RNA-seq.
(XLS)

Table S3 Known zebrafish genes that form the core clock. The temporal pattern of each gene, as determined using RNA-seq, is compared to previously reported experimental data.
(DOCX)

Table S4 Known zebrafish clock-controlled genes that are considered to form accessory loops of the molecular circadian oscillator. The temporal pattern of each gene, as determined using RNA-seq, is compared to previously reported experimental data.
(DOCX)

Table S5 DAVID analysis of gene functional annotations.
(XLS)

Table S6 DAVID analysis: a) genes that belong to the glycolysis pathway and b) genes that belong to the oxidation-reduction pathway.
(XLS)

Text S1 Supplementary Methods.
(DOC)
