# Supplementary figures and images for: Systematic Identification of Rhythmic Genes Reveals camk1gb as a New Element in the Circadian Clockwork
Source: PLoS Genet. 2012 Dec 20;8(12):e1003116. doi: 10.1371/journal.pgen.1003116 (PMC3527293; doi:10.1371/journal.pgen.1003116)

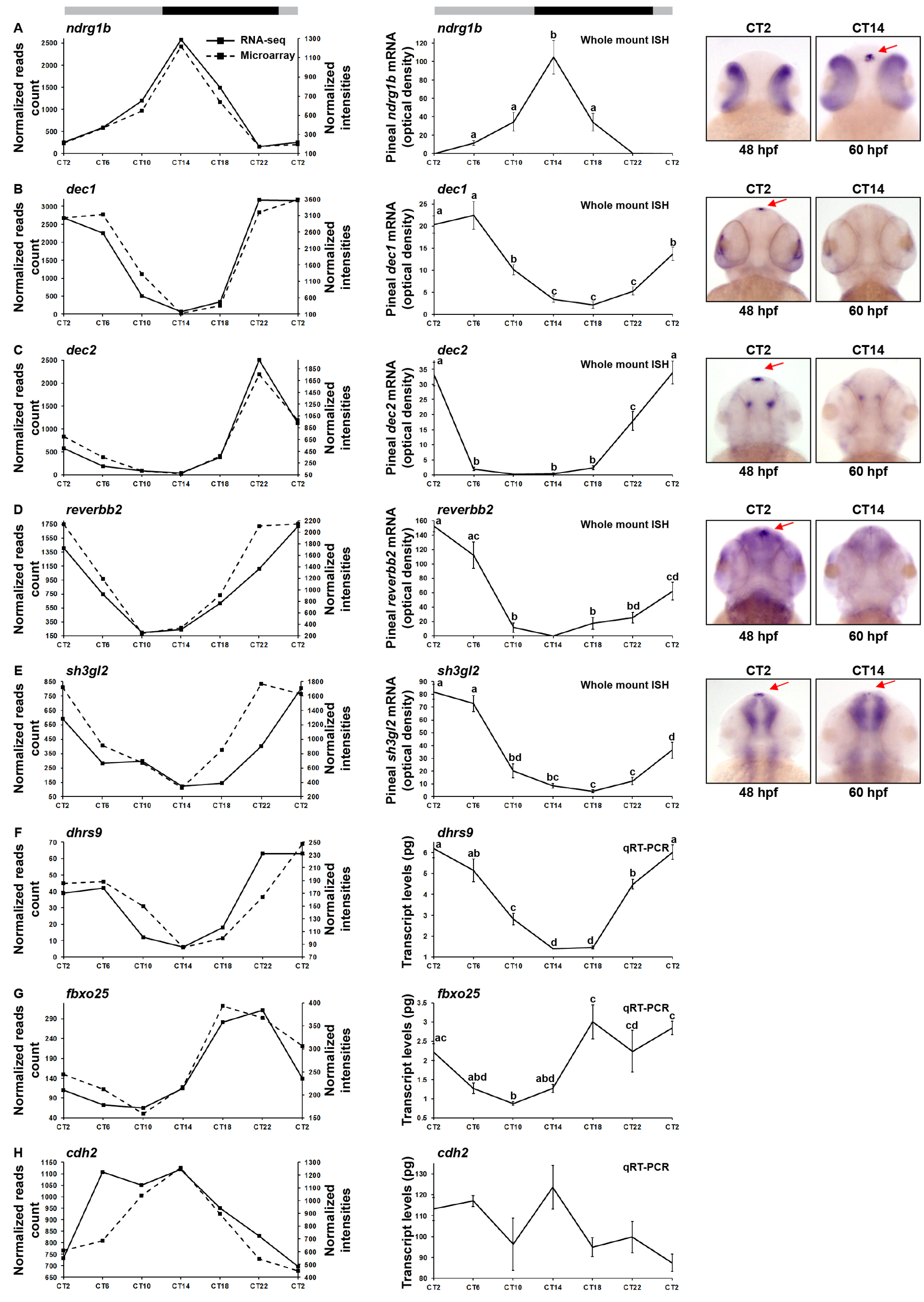

Supplement: Figure S1 — Whole-mount ISH and qRT-PCR validations. The circadian expression of several genes was validated using whole mount ISH on embryos at the age of 48–72 hours (A–E, right curves) and qRT-PCR on adult pineal glands (F–H, right curves). Left curves represent the circadian profile of each gene as obtained by RNA-seq (solid line, left vertical bar) and DNA-microarray (dashed line, right vertical bar). Representative pictures of embryos heads (dorsal view), subjected to whole mount ISH for ndrg1b, dec1, dec2, reverbb2 and sh3gl2 at CT2 and CT14, are presented at the rightmost side of A–E. The pineal gland is indicated by a red arrow. Different letters represent statistical differences in mRNA levels as determined by one-way ANOVA followed by a Tukey test (P-value<0.05). Note that cdh2 was not validated as circadian (H). Error bars represent SE (n = 10–15). CT = circadian time. Gray and black bars represent subjective day and subjective night, respectively. Whole mount ISH validation of camk1gb is given in Figure 3. (TIF) [file pgen.1003116.s001.tif]

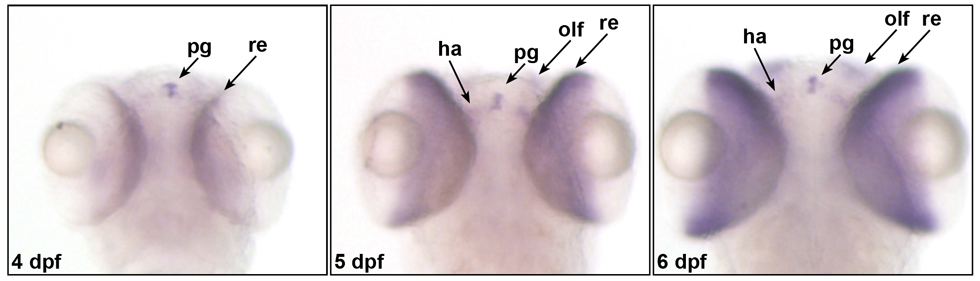

Supplement: Figure S2 — camk1gb expression at later larval stages. camk1gb expression on days 4–6 post fertilization is enhanced in the pineal gland (pg) and expends to the retina (re), habenula (ha) and olfactory bulbs (olf) as detected by whole mount ISH. (TIF) [file pgen.1003116.s002.tif]

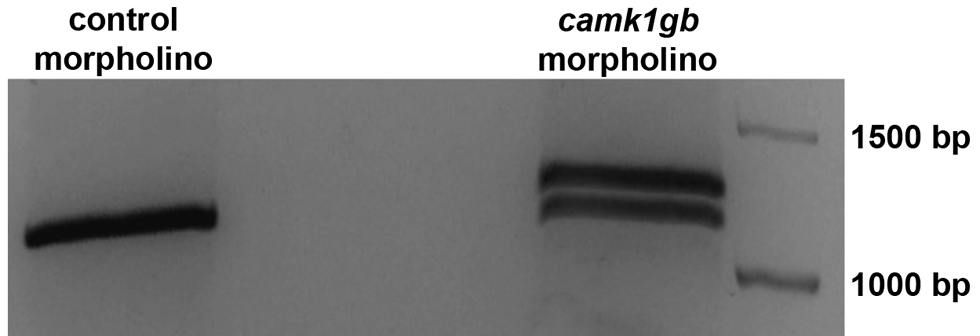

Supplement: Figure S3 — PCR analysis of camk1gb following camk1gb morpholino injection. camk1gb morpholino injection changed the normal splicing of camk1gb mRNA, leading to an insertion of intron5 (right lane) which adds a premature stop codon. Injection of control morpholino had no effect on camk1gb splicing (left lane). (TIF) [file pgen.1003116.s003.tif]

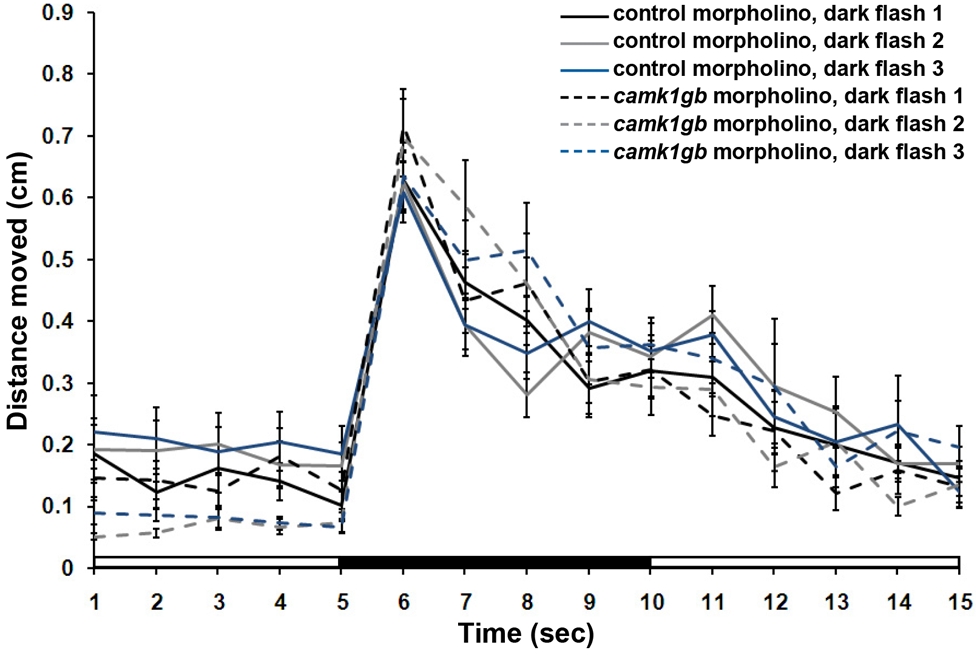

Supplement: Figure S4 — Locomotor activity levels under 3 dark flash stimuli. On day 6 post fertilization, control morpholino (solid line) and camk1gb morpholino (dashed line) injected larvae (n = 24) were subjected to 3 dark flashes (black, gray and blue lines) of 5 sec each during the light phase. Activity was measured as the average distance moved in 1 sec time bins. Error bars represent SE (n = 24). White and black horizontal boxes represent light phase and dark flash, respectively. (TIF) [file pgen.1003116.s004.tif]

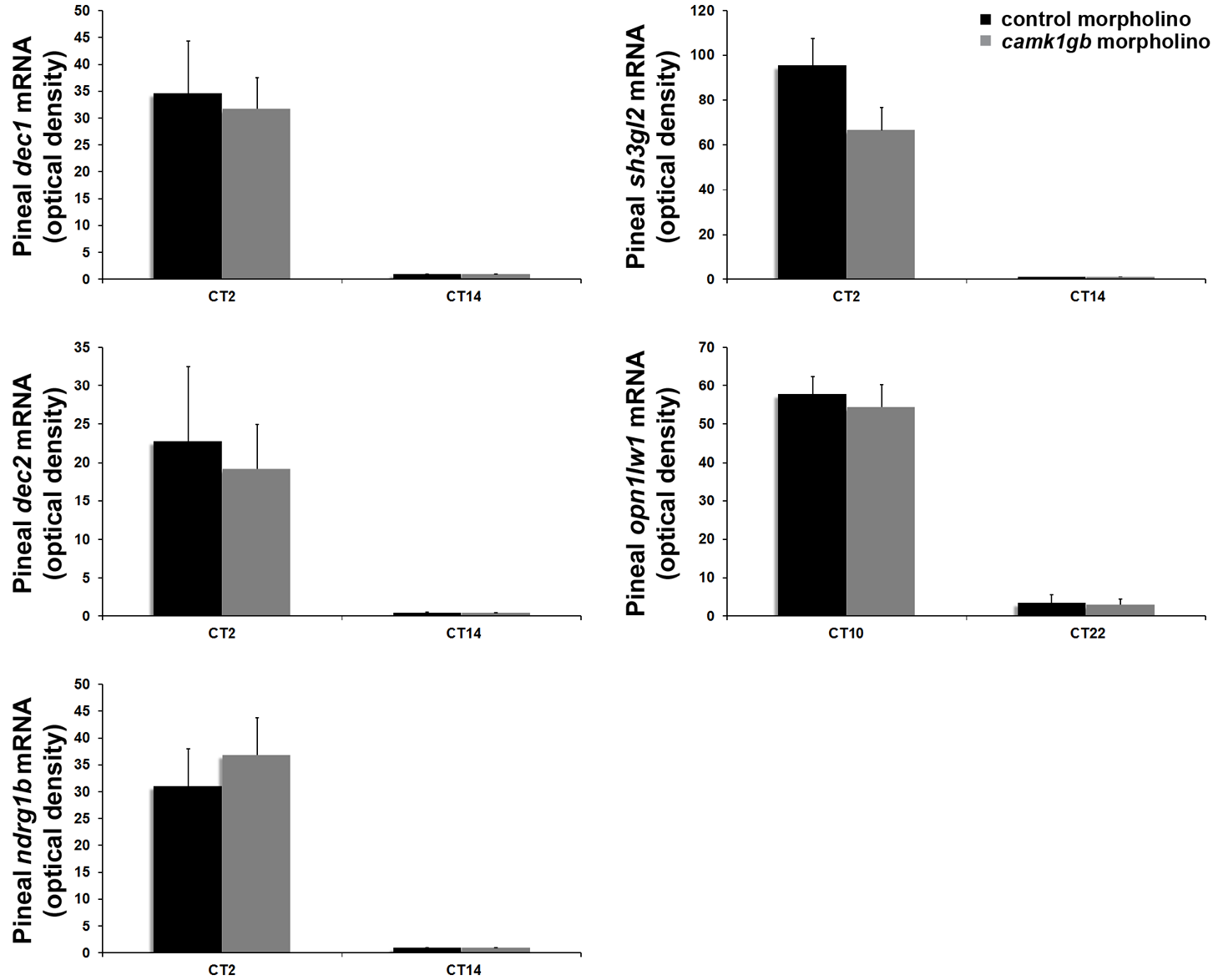

Supplement: Figure S5 — Effect of camk1gb knockdown on the expression of known clock-controlled genes. Zebrafish embryos injected with either control morpholino (black bar) or camk1gb morpholino (gray bar) were subjected to DD during their third day of development and sampled at the peak and the nadir of their rhythm. Pineal mRNA levels of dec1, dec2, ndrg1l, sh3gl2 and opn1lw1 were determined by whole mount ISH. No statistically significant differences were observed (two-tailed t-test). Error bars represent SE (n = 10–15); CT = circadian time. (TIF) [file pgen.1003116.s005.tif]

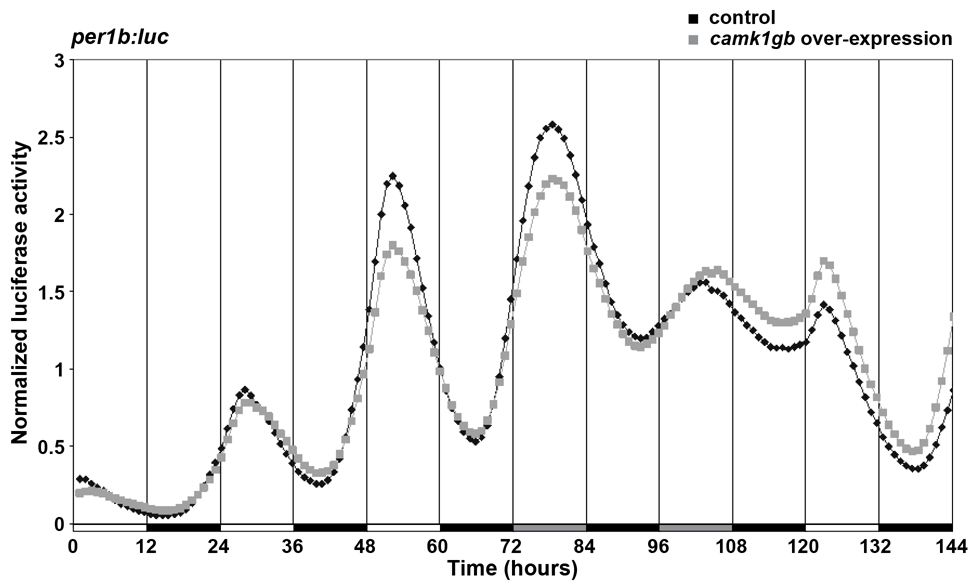

Supplement: Figure S6 — The effect of camk1gb over-expression on the core clock marker per1b. The zebrafish photosensitive Pac-2 cell line were transiently co-transfected with camk1gb and per1b:luciferase constructs. Bioluminescence was monitored under LD and DD conditions. No significant differences were found in the reporter construct expression as a result of camk1gb over-expression. White and black bars show the light and dark periods, respectively. Grey bars represent subjective day. (TIF) [file pgen.1003116.s006.tif]

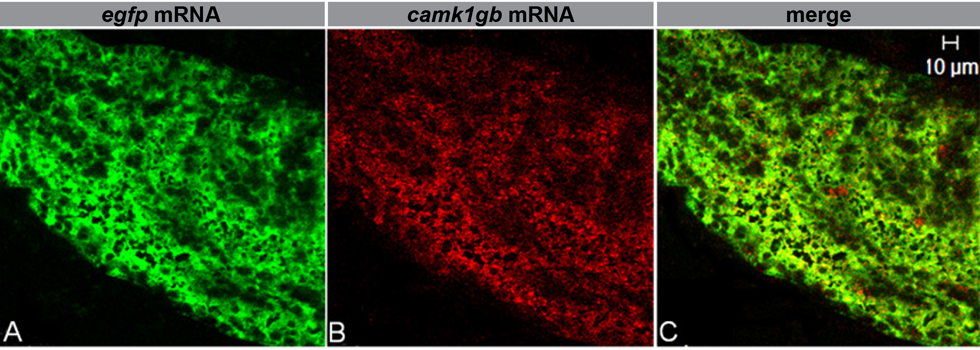

Supplement: Figure S7 — camk1gb expression in the adult pineal gland of transgenic zebrafish, Tg(aanat2:EGFP)Y8. Double fluorescent in situ hybridization for egfp mRNA (A, green) and camk1gb mRNA (B, red) in adult pineal glands, reveals co-expression of camk1gb and the aanat2:EGFP transgene (C, merged image). Scale bar = 10 µm. (TIF) [file pgen.1003116.s007.tif]

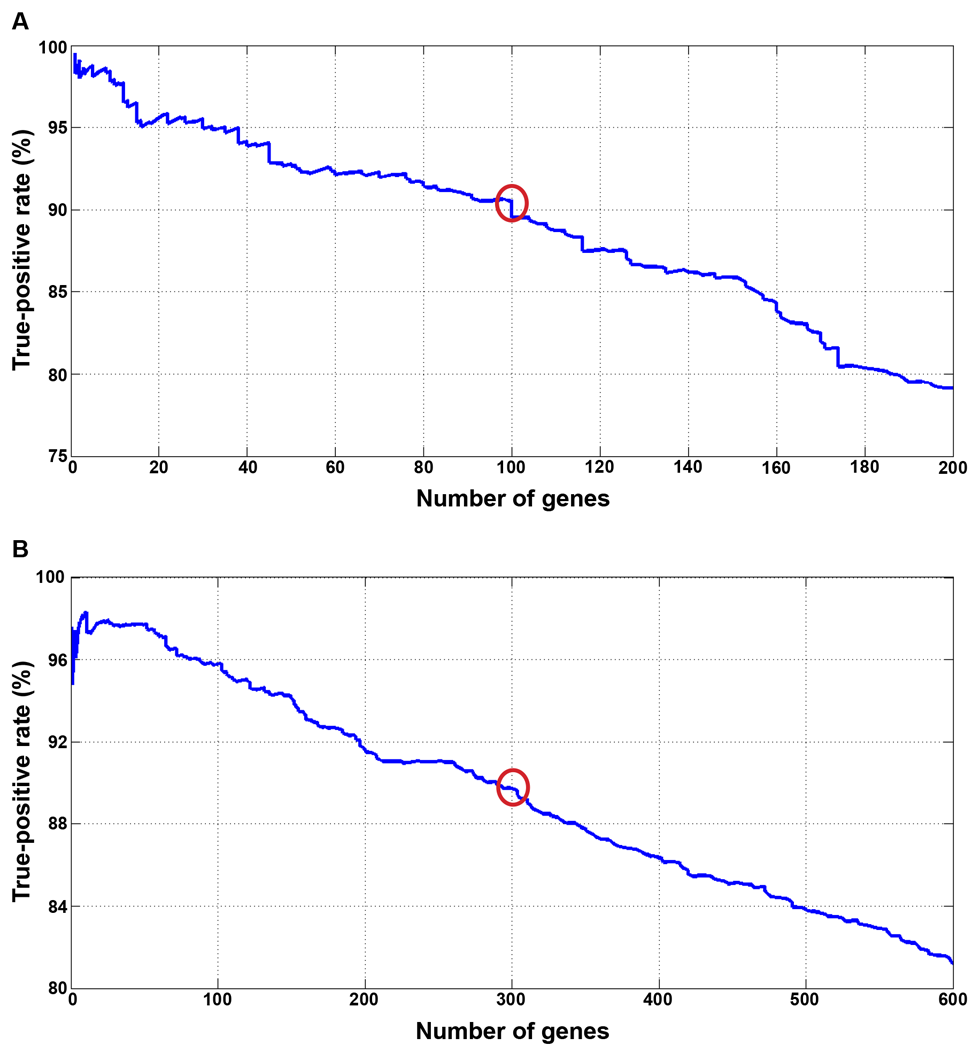

Supplement: Figure S8 — True-positive rate as a function of the number of circadian transcripts detected. A) DNA microarray experiment and B) RNA-Seq experiment. Red circles mark the size of the chosen list of transcripts. (TIF) [file pgen.1003116.s008.tif]
