# Supplementary material for: Systematic Identification of Rhythmic Genes Reveals camk1gb as a New Element in the Circadian Clockwork
Source: PLoS Genet. 2012 Dec 20;8(12):e1003116. doi: 10.1371/journal.pgen.1003116 (PMC3527293; doi:10.1371/journal.pgen.1003116)
Supplement: Table S3 — Known zebrafish genes that form the core clock. The temporal pattern of each gene, as determined using RNA-seq, is compared to previously reported experimental data. (DOCX) [file pgen.1003116.s011.docx]

**Table S3:** Known zebrafish genes that form the core clock. The temporal pattern of each gene, as determined using RNA-seq, is compared to previously reported experimental data.

| Previously reported experimental data | | | |  | RNA-seq of the pineal gland | | | Clock genes^1^ |
| --- | --- | --- | --- | --- | --- | --- | --- | --- |
| References | Tissue |  | Expression peaks |  | Expression peaks | Temporal pattern | |  |
| [1] | Muscle | CT2 (DD) | |  | CT2 | Circadian | | *cry1a* |
| [2] | Eye | CT1-CT5 (LD, DD) | |  |  |  | |  |
| [2] | Eye | CT13 (LD) | |  | CT22-CT2 | Circadian | | cry1b |
| [2] | Eye | CT13-CT15 (DD) | |  | CT14 | Circadian^2^ | | *cry2a* |
| [1] | Muscle | CT14 (DD) | |  |  |  | |  |
| [3] | Larva | CT12 (LD) | |  |  |  | |  |
| [2] | Eye | CT13-CT15 (DD) | |  | CT14 | Circadian | | *cry2b* |
| [1] | Muscle | CT14 (DD) | |  |  |  | |  |
| [1] | Muscle | CT23 (DD) | |  | CT22 | Circadian^2^ | | *cry3* |
| [3] | Larva | CT21 (LD) | |  |  |  | |  |
| [2] | Eye | CT9 (LD,DD) | |  | CT10 | Circadian | | *cry4* |
| [1] | Muscle | CT23 (DD) | |  | CT22 | Circadian | | *per1a* |
| [1] | Muscle | CT1 (DD) | |  | CT22-CT2 | Circadian | | *per1b* |
| [4] | Brain, larva | CT22-24 (LD) | |  |  |  | |  |
| [5] | Larva | Not circadian | |  |  | Not circadian | | *per2* |
| [1] | Muscle | CT1 (DD) | |  | CT2 | Circadian | | *per3* |
| [6] | CNS, retina | CT0-CT4 (DD,LD) | |  |  |  | |  |
| [7] | Adult pineal gland | CT9-CT15(LD) | |  | CT10 | Circadian^2^ | | *bmal1a* |
| [8] | Eye | CT13-CT15 (LD, DD) | |  |  |  | |  |
| [8] | Eye | CT9-CT13 (LD, DD) | |  | CT10 | Circadian^3^ | | *bmal1b* |
| [7] | Adult pineal gland | CT15 (LD) | |  | CT10-CT14 | Circadian | | *bmal2* |
| [8] | Brain, eye | CT9-CT15 (DD,LD) | |  |  |  | |  |
| [9],[8] | Adult pineal gland, eye | CT9-CT15 (DD, LD) | |  | CT10 | Circadian | | *clock1a* |
| [1] | Muscle | CT14 (DD) | |  | CT10 | Circadian | *clock1b* | |
| [8] | Eye | CT13 (LD) | |  |  |  |  | |
| [8] | Eye | CT9-CT13 (DD, LD) | |  | CT14 | Circadian | | *clock2* |

^1^Gene nomenclature is according to Vatine et al. [10]

^2^These genes were also identified using the DNA microarray data

^3^*bmal1b* was identified as circadian by Fourier analysis with 85% true-positives

References:

1.Amaral IPG, Johnston IA (2012) Circadian expression of clock and putative clock-controlled genes in skeletal muscle of the zebrafish. Am J PhysiolRegulIntegr Comp Physiol 302: R193–R206.

2.Kobayashi Y, Ishikawa T, Hirayama J, Daiyasu H, Kanai S, et al. (2000) Molecular analysis of zebrafishphotolyase/cryptochrome family: two types of cryptochromes present in zebrafish. Genes Cells 5: 725–738.

3.Lahiri K, Vallone D, Gondi SB, Santoriello C, Dickmeis T, et al. (2005) Temperature regulates transcription in the zebrafish circadian clock. PLoSBiol 3: e351.

4.Vallone D, Gondi SB, Whitmore D, Foulkes NS (2004) E-Box function in a period gene repressed by light. ProcNatlAcadSci USA 101: 4106–4111.

5.Ziv L, Levkovitz S, Toyama R, Falcon J, Gothilf Y (2005) Functional development of the zebrafish pineal gland: light‐induced expression of period2 is required for onset of the circadian clock. Journal of Neuroendocrinology 17: 314–320.

6.Delaunay F, Thisse C, Marchand O, Laudet V, Thisse B (2000) An inherited functional circadian clock in zebrafish embryos. Science 289: 297–300.

7.Cermakian N, Whitmore D, Foulkes NS, Sassone-Corsi P (2000) Asynchronous oscillations of two zebrafish CLOCK partners reveal differential clock control and function. ProcNatlAcadSci USA 97: 4339–4344.

8.Ishikawa T, Hirayama J, Kobayashi Y, Todo T (2002) Zebrafish CRY represses transcription mediated by CLOCK‐BMAL heterodimer without inhibiting its binding to DNA. Genes to Cells 7: 1073–1086.

9.Whitmore D, Foulkes NS, Strähle U, Sassone-Corsi P (1998) Zebrafish Clock rhythmic expression reveals independent peripheral circadian oscillators. Nat Neurosci 1: 701–707.

10.Vatine G, Vallone D, Gothilf Y, Foulkes NS (2011) It’s time to swim! Zebrafish and the circadian clock. FEBS Letters 585: 1485–1494.
