# Supplementary material for: Systematic Identification of Rhythmic Genes Reveals camk1gb as a New Element in the Circadian Clockwork
Source: PLoS Genet. 2012 Dec 20;8(12):e1003116. doi: 10.1371/journal.pgen.1003116 (PMC3527293; doi:10.1371/journal.pgen.1003116)
Supplement: Table S4 — Known zebrafish clock-controlled genes that are considered to form accessory loops of the molecular circadian oscillator. The temporal pattern of each gene, as determined using RNA-seq, is compared to previously reported experimental data. (DOCX) [file pgen.1003116.s012.docx]

**Table S4**: Known zebrafishclock-controlled genes that are considered to form accessory loops of the molecular circadian oscillator. The temporal pattern of each gene, as determined usingRNA-seq, is compared to previously reported experimental data.

| Previously reported experimental data | | | |  | RNA-seq of the pineal gland | | Clock controlled genes |
| --- | --- | --- | --- | --- | --- | --- | --- |
| References | Tissue |  | Expression peak |  | Expression peak | Temporal pattern |  |
| [1] | Muscle | CT6 (DD) | |  | Not identified | Not identified | *roraa* |
| [1] | Muscle | CT22 (DD) | |  | CT2 | Circadian | *rorab* |
|  |  | Not reported | |  | CT6-CT10 | Circadian | *rorca* |
| [1] | Muscle | CT22 (DD) | |  | CT22 | Circadian | *nr1d1 (reverba)* |
| [1] | Muscle | CT22 (DD) | |  | Not identified | Not identified | *nr1d2a (reverbb1)* |
| [1] | Muscle | CT22-CT2 (DD) | |  | CT22-CT2 | Circadian^1^ | *nr1d2b (reverbb2)* |
|  |  | Not reported | |  | CT10 | Circadian | *nfil3 (e4pb4)* |
| [2] | Embryos pineal gland | CT10-CT18 (DD) | |  | CT10 | Circadian | *nfil3-2 (e4pb4-2)* |
| [2] | Embryos pineal gland | CT10-CT14 (DD) | |  | CT10 | Circadian^1^ | *nfil35 (e4pb4-5)* |
| [2] | Embryos pineal gland | CT2 (LD) | |  | CT6 | Circadian | *nfil3-6 (e4pb4-5)* |
| [2] | Embryos pineal gland | CT22 (DD) | |  | CT22 | Circadian^1^ | *dbpa* |
| [2] | Embryos pineal gland | CT22 (DD) | |  | CT22 | Circadian | *dbpb* |
| [3] | BRF41 cells | CT2-CT6 (DD) | |  | CT22 | Circadian^1^ | *bhlhe40 (dec1)* |
| [3] | BRF41 cells | CT22-CT2 (DD) | |  | CT22 | Circadian^1^ | *bhlhe41 (dec2)* |

^1^These genes were also identified using the DNA microarray data

References:

1. Amaral IPG, Johnston IA (2012) Circadian expression of clock and putative clock-controlled genes in skeletal muscle of the zebrafish. Am J PhysiolRegulIntegr Comp Physiol 302: R193–R206.

2. Ben-Moshe Z, Vatine G, Alon S, Tovin A, Mracek P, et al. (2010) Multiple PAR and E4BP4 bZIP transcription factors in zebrafish: diverse spatial and temporal expression patterns. ChronobiolInt 27: 1509–1531.

3. Abe T, Ishikawa T, Masuda T, Mizusawa K, Tsukamoto T, et al. (2006) Molecular analysis of Dec1 and Dec2 in the peripheral circadian clock of zebrafish photosensitive cells. BiochemBiophys Res Commun 351: 1072–1077.
