# Supplementary material for: Systematic Identification of Rhythmic Genes Reveals camk1gb as a New Element in the Circadian Clockwork
Source: PLoS Genet. 2012 Dec 20;8(12):e1003116. doi: 10.1371/journal.pgen.1003116 (PMC3527293; doi:10.1371/journal.pgen.1003116)
Supplement: Text S1 — Supplementary Methods. (DOC) [file pgen.1003116.s015.doc]

**Systematic identification of rhythmic genes reveals *camk1gb* as a new element in the circadian clockwork**

Adi Tovin^1,*^, Shahar Alon^1,2,*^, Zohar Ben-Moshe^1^, Philipp Mracek^3^, Gad Vatine^1^, Nicholas S. Foulkes^3^, Jasmine Jacob-Hirsch^4^, Gideon Rechavi^4^, Reiko Toyama^5^, Steven L. Coon^6^, David C. Klein^6^, Eli Eisenberg^2,7^ and Yoav Gothilf^1,2^

1. Department of Neurobiology, George S. Wise Faculty of Life Sciences, Tel Aviv University, Tel Aviv 69978, Israel
2. Sagol School of Neuroscience, Tel Aviv University, Tel Aviv 69978, Israel
3. Institute of Toxicology and Genetics, Karlsruhe Institute of Technology, Eggenstein 76344, Germany
4. Cancer Research Center, Sheba Medical Center, Tel Hashomer and Sackler School of Medicine, Tel Aviv University, Tel Aviv 69978, Israel
5. Laboratory of Molecular Genetics, Eunice Kennedy Shriver National Institute of Child Health and Human Development, National Institutes of Health, Bethesda, MD 20892, USA
6. Program in Developmental Endocrinology and Genetics, Eunice Kennedy Shriver National Institute of Child Health and Human Development, National Institutes of Health, Bethesda, MD 20892, USA
7. Raymond and Beverly Sackler School of Physics and Astronomy, Tel Aviv University, Tel Aviv 69978, Israel

*These authors contributed equally to this work.

Correspondence should be addressed to: Yoav Gothilf, [yoavg@tauex.tau.ac.il](mailto:yoavg@tauex.tau.ac.il) and Eli Eisenberg, [elieis@post.tau.ac.il](../../../../D:/My%20Documents/Downloads/elieis@post.tau.ac.il)

**Supplementary Methods**

**Assessing the quality of the sequencing libraries**

The quality of the sequencing libraries was assessed and compared to the benchmark library described in Levin et al. [1]. The tests included: a) Evenness of coverage: the average of the coefficient of variation of gene coverage for the top 50% expressed genes. The libraries had average coefficient of variation of ~0.55, even lower than the benchmark library (~0.75). b) Continuity of coverage: the number of segments into which each known transcript was broken, where a break is defined as a stretch of at least five bases without read coverage. The measurements were averaged across all genes, weighted by the relative expression of each gene. Our libraries had on average ~2 segments for each mRNA, lower than the benchmark library (~3). c) Coverage at the ends of the coding region: the number of genes with complete coverage at the ends of the coding region (10 bases before the start codon and 10 bases after the stop codon). In our libraries ~55% of the known coding genes were covered at the ends of the coding region. These numbers are similar to the numbers of the benchmark library (~60% in each side). Thus, all tests indicate that the quality of our libraries meets the benchmark library described by Levin et al. [1].

**Normalization of the sequencing libraries**

The 12 mRNA profiles (i.e. the number of reads aligned against each RefSeq gene) corresponding to the 12 time points were normalized. We tested two normalization methods on the logarithmically transformed dataset: 1) Quantile normalization as described for the DNA microarrays datasets in the Methods. 2) A modification of the TMM normalization method [2] in which the mRNA profiles were scaled such that the log-fold changes of all the mRNAs are distributed around zero (after trimming the higher and lower quartiles of the log-fold changes). The scaling factor is thus set so that the trimmed mean of log-folds vanish. The mean is weighted using the inverse standard deviation, as estimated from Poisson distribution of counts [3]. These two normalization methods gave similar results; the former was used in the analysis.

**Validation of normalization and transcripts filtering**

To determine whether our normalization scheme properly corrects for different RNA levels and other technical differences between samples, we examined the expression levels of two known housekeeping genes, beta actin 2 (*actb2*) and ribosomal protein L3 (*rpl3*). The coefficients of variation (CV) of their normalized expression levels along the 12 time points were less than 1% for both the DNA microarrays and RNA-seq data. Therefore, the expression data of these two housekeeping genes is consistent with the claim that the normalization scheme properly accounted for varying RNA levels between samples. Transcripts with low maximum expression value (i.e. their highest level over all time points is in the lower quartile of all transcripts) were not included in the Fourier analysis.

**Whole mount ISH**

Six DNA fragments containing the coding region of the following genes: *camk1gb, reverbb2, dec1, dec2, ndrgl1 and sh3gl2* were amplified using the following primers: *camk1gb* primers: 5'- atgggccgaaaggagggcgaatat-3' and 5'- tcacatgacagagcaaacaccagtctgc-3'; *reverbb2* primers: 5'- caagagatgctgtgcgttttg-3' and 5'-tgcgttgcttttctcgtttg-3'; *dec1* primers: 5'-cgcggatccaaatggagaggattaccag-3' and 5'-cgcgaattcgtctttggtttccagg-3'; *dec2* primers: 5'- atggaagaaagaatatcgagaatgcagaaca-3' and 5'- ttatgtaccatcattgtcatttctttgggg-3'; *ndrgl1* primers: 5'- atggtactggaggactccgagagca-3' and 5'-tcagcaggtcaactcgacggaaact-3'; *sh3gl2* primers: 5'-atgtctgtagcgggactgaagaaacaattc-3' and 5'-ttaatgaggcaaagctaccaggatatccac-3'. The PCR products were cloned into a pGEM-T Easy vector (Promega). These clones were linearized and served as templates for *in vitro* synthesis of digoxygenin (DIG)-labeled antisense riboprobes (DIG RNA labeling kit; Roche) by SP6 or T7 RNA polymerases according to the orientation of each insert. Probes were used at a concentration of 1ng/µl. Embryos and larvae were sampled at different circadian time points. In order to determine rhythmicity of the studied genes, embryos were kept under LD cycles during the first 2 days of development, then transferred to DD, collected at 4-hr intervals throughout a daily cycle (n=10-15 embryos/time point), and subjected to whole mount ISH as described [4]. The sampling time-points corresponded to the following circadian times (CTs 2, 6, 10, 14, 18, 22 and 2b). ISH-stained specimens were fixed in 4% paraformaldehyde and stored in 100% methanol. For image analysis, the stained larvae were placed in 70% glycerol and photographed from dorsal view using an Olympus dissecting microscope (SZX12, Olympus) equipped with digital camera (DP70, Olympus). For every tested gene, the average signal intensity in the pineal gland was measured using ImageJ software (National Institutes of Health, Bethesda, Maryland). Time-dependent statistical differences in mRNA levels were determined by one-way ANOVA followed by a Tukey test.

**Quantitative real time PCR (qRT-PCR)**

mRNA levels of *fbxo25, dhrs9* and *cdh2* were measured using qRT-PCR. Adult (0.5-1.5 years old) transgenic zebrafish, *Tg(aanat2:EGFP)^Y8^* were maintained under LD conditions, transferred to DD and pineal glands were removed under a fluorescent dissecting microscope at 4-hr intervals throughout a daily cycle (CTs 2, 6, 10, 14, 18, 22 and 2b). At each time point, three pools of six pineal glands were collected. Total RNA was extracted using an RNeasy Lipid Tissue Mini Kit (QIAGEN) according to the manufacturer's instructions. The mRNA was reverse-transcribed using a High Capacity cDNA Reverse Transcription Kit (Applied Biosystems). mRNA levels were determined by absolute quantification using the ABI Prism 7300 thermocycler (Applied Biosystems), according to manufacturer’s protocol. Triplicates of each cDNA sample were PCR amplified by a SYBR Green PCR Master Mix (Applied Biosystems) and specific primers: *fbxo25* primers: 5'-atggcagcagcagctcaata-3' and 5'-ccgtttgaaacttgctttgga-3'; *dhrs9* primers: 5'-gtcaaggatgagtacggcagc-3' and 5'-ggccgtcagctttgtcttgt-3'; *cdh2* primers: 5'-ccgtgagaaggtgcctcagt-3' and 5'-tccatatcggtagcctggatg-3'. The relative copy number of each gene in each sample was determined by comparing to a recombinant plasmid standard curve (ranging 0.5ng - 0.5*10^-6^ ng) containing the specific cDNA insert. Differences in mRNA levels among time points of each gene were determined by one-way ANOVA followed by a Tukey test.

**Morpholino design and knockdown experiments**

One cell stage embryos were injected with morpholino-modified antisense oligonucleotides (Gene Tools) at approximately 2nl volume (1 mM each). The following morpholinos were used: 1) Gene Tools standard control morpholino (5′-ctcttacctcagttacaatttata-3′); 2) *camk1gb* morpholino (5′-aaactctgatgagaccgcagcagtt-3′) which was designed to block splicing between intron 4 and exon 5; The efficiency of *camk1gb* knockdown was determined by analyzing the sequence and amount of un-spliced vs. spliced *camk1gb* transcript. Morpholino-injected embryos were sampled at 60 hours post-fertilization and total RNA was extracted using EZ-RNA Isolation Kit (Biological Industries). cDNA was reverse transcribed using M-MLV reverse transcriptase (Promega) and oligo dT primers. To confirm *camk1gb* morpholino efficiency, PCR amplification was conducted using the following sets of primers: 5'-gggccgaaaggagggcgaatatgaatgg-3' and 5'-aaggcttgcttccacttggacttggcg-3', ensuring the formation of a new transcript (Figure S3). The PCR products were cloned into pGEM-T Easy vector (Promega) and sequenced, revealing the insertion of intron 5 into the *camk1gb* coding sequence and the inclusion of an early stop codon into the protein sequence. It should be noted that the injected morpholino did not completely abolish the endogenous *camk1gb* (Figure S3). Using the above procedure, the efficiency of *camk1gb* knockdown was validated until day 8 post-fertilization.

To examine the effect of *camk1gb* knockdown on *aanat2* expression in the pineal gland, *camk1gb* morpholino injected embryos were subjected to whole mount ISH as described above. Statistical differences in *aanat2* mRNA levels between *camk1gb* morpholino and control morpholino injected embryos were determined by two-tailed *t*-test with Bonferroni correction (Figure 5). To confirm normal development of the pineal gland, the effect of *camk1gb* morpholino injection on *otx5* mRNA expression was tested by whole mount ISH at CT18, as described above.

The effect of the *camk1gb* morpholino injection on *dec1, dec2, sh3gl2, opn1lw1* and *ndrg1*b mRNA was tested by whole mount ISH, at the peak and the nadir of their rhythm (Figure S5). The following primers were used for constructing *opn1lw1* antisense riboprobes: 5'- atggcagagcattggggagatgc-3' and 5'- ttatgcaggagccacagaagacacttctg-3'. Statistical differences between *camk1gb* morpholino and control morpholino treated embryos were tested (separately) for the maximum expression and minimum expression in each of the examined genes (two-tailed *t*-test with Bonferroni correction, n=10).

**Transfection of *camk1gb* into the Pac-2 cell line**

Transient co-transfection of the Pac-2 cell line with *camk1gb* and *per1b*:luciferase constructs was performed as previously described [5]. *camk1gb* over-expression was conducted using the same construct as described in the Methods section 'Rescue experiments'. The effect of the co-transfection was assessed by bioluminescence monitoring during three light-dark cycles followed by two days of constant darkness and two days of dark-light cycles (Figure S6). A knockdown experiment was not considered in Pac-2 cells because the expression level of *camk1gb* in this cell line is in the lower decile in the distribution of all the genes, as observed by DNA microarray analysis of the Pac-2 cell line (Probe Set ID Dr.9852.1.A1_at) [6], suggesting that this gene is not expressed at all in this cell line. For comparison, *camk1gb* expression levels in the pineal gland were in the upper decile in the distribution of all the genes, making the knockdown approach adequate for the *in vivo* experiments.

**Double ISH of *camk1gb* and *aanat2*:EGFP**

To determine if *camk1gb* is expressed within the photoreceptor cells of the pineal gland, double fluorescence ISH of *camk1gb* and *egfp* in adult (1 year old) transgenic zebrafish, *Tg(aanat2:EGFP)^Y8^*, was done. These transgenic zebrafish express *egfp* within the photoreceptor cells [7]. For double fluorescence ISH we followed the protocol of Machluf and Levkowitz [8]. Briefly, digoxigenin (DIG)-labeled *camk1gb* and fluorescein-labeled *egfp* antisense riboprobes (2 ng/µl) were simultaneously hybridized. Next, anti-fluorescein-POD antibody (Roche) and anti-digoxigenin-AP antibody (Roche) were simultaneously incubated over-night. *camk1gb* mRNA was visualized by an enzymatic reaction using Fast Red substrate (Roche). *egfp* mRNA was subsequently visualized using TSA™ Plus Fluorescein System (Perkin-Elmer). Imaging was done using confocal microscopy (Figure S7).

**Supplementary References**

1. Levin JZ, Yassour M, Adiconis X, Nusbaum C, Thompson DA, et al. (2010) Comprehensive comparative analysis of strand-specific RNA sequencing methods. Nat Methods 7: 709–715.

2. Robinson MD, Oshlack A (2010) A scaling normalization method for differential expression analysis of RNA-seq data. Genome Biol 11: R25.

3. Alon S, Vigneault F, Eminaga S, Christodoulou DC, Seidman JG, et al. (2011) Barcoding bias in high-throughput multiplex sequencing of miRNA. Genome Res 21: 1506-1511.

4. Ziv L, Levkovitz S, Toyama R, Falcon J, Gothilf Y (2005) Functional development of the zebrafish pineal gland: light‐induced expression of period2 is required for onset of the circadian clock. Journal of Neuroendocrinology 17: 314–320.

5. Cavallari N, Frigato E, Vallone D, Fröhlich N, Lopez-Olmeda JF, et al. (2011) A blind circadian clock in cavefish reveals that opsins mediate peripheral clock photoreception. PLoS Biol 9: e1001142.

6. Weger BD, Sahinbas M, Otto GW, Mracek P, Armant O, et al. (2011) The light responsive transcriptome of the zebrafish: function and regulation. PLoS ONE 6: e17080.

7. Gothilf Y, Coon SL, Toyama R, Chitnis A, Namboodiri M a. A, et al. (1999) Zebrafish serotonin N-acetyltransferase-2: marker for development of pineal photoreceptors and circadian clock function. Endocrinology 140: 4895–4903.

8. Machluf Y, Levkowitz G (2011) Visualization of mRNA Expression in the zebrafish embryo. In: Gerst JE, editor. RNA detection and visualization. Methods in Molecular Biology. Humana Press. pp. 83–102.
